# An 8-Year-Old Female with Giardiasis-Associated Henoch–Schönlein Purpura: A Case Report and Literature Review

**DOI:** 10.3390/reports9010005

**Published:** 2025-12-22

**Authors:** Konstantinos Miliordos, Dimitrios Kapnisis, Christodoulos Chatzigrigoriadis, Emmanouil Koufopoulos, Sokratis Tsantiris, Aris Bertzouanis, Eirini Kostopoulou, Despoina Gkentzi

**Affiliations:** 1 Department of Pediatrics, University General Hospital of Patras, Patras Medical School, 26504 Rio, Greece; mhlcon95@gmail.com (K.M.); kapn.dim@hotmail.com (D.K.); aris_berd@riseup.net (A.B.); eirini.kost@gmail.com (E.K.); gkentzid@upatras.gr (D.G.); 2 School of Medicine, University of Patras, 26504 Patras, Greece; up1084128@ac.upatras.gr; 3 Forensic Service of Piraeus, Ministry of Justice, 18535 Piraeus, Greece; s.tsantiris@gmail.com

**Keywords:** *Giardia lamblia*, giardiasis, IgA vasculitis, purpura, post-infectious disorders, exanthema, diarrhea, arthritis, arthralgia, leukocytoclastic vasculitis

## Abstract

**Background and Clinical Significance:** Henoch–Schönlein purpura (HSP), also known as Immunoglobulin A (IgA) vasculitis (IgAV), is a common systemic vasculitis in children characterized by palpable purpura, abdominal pain, and joint and kidney involvement. While respiratory tract viral or bacterial infections are the most common causes of HSP, parasitic infections, such as giardiasis, are occasionally reported. *Giardia lamblia* is the most common parasite infecting humans and a major cause of infectious diarrhea, which can lead to post-infection complications. To our knowledge, this is the first report in Greece describing a pediatric patient with HSP secondary to giardiasis. A review of pediatric HSP cases caused by parasitic infections is also included. **Case presentation**: An 8-year-old girl presented with a purpuric rash, joint tenderness, severe abdominal pain, and bloody diarrhea, raising suspicion of HSP. Laboratory tests revealed elevated IgA levels, and stool analysis tested positive for *Giardia lamblia* antigen. The diagnosis of HSP secondary to giardiasis was confirmed, and the patient was successfully treated with supportive care, metronidazole, and corticosteroids. **Conclusion**: This case report and literature review highlight parasitic infections as an underrecognized but important trigger of pediatric HSP. Although giardiasis is linked to various post-infectious complications, its association with HSP is rarely reported. Pediatricians should maintain a high level of suspicion for underlying infectious diarrhea, such as giardiasis, in patients with HSP, especially in children with prominent gastrointestinal symptoms. Early recognition can reduce complications and facilitate faster recovery. Further research is needed for the immunopathogenic mechanisms linking parasitic infections and HSP in children.

## 1. Introduction and Clinical Significance

Henoch–Schönlein purpura (HSP), also known as IgA vasculitis (IgAV), is an IgA-mediated inflammatory disease affecting the small blood vessels, especially in the skin, joints, gastrointestinal tract, and kidneys [1,2,3,4,5,6,7]. HSP typically affects children, with an estimated incidence of 3 to 26.7 patients per 100,000 children, but this varies among nationalities [1,3,4,5,6,7,8,9]. HSP is typically preceded by an acute infectious illness, most commonly a respiratory tract infection, followed by infectious diarrhea, skin infections, and urinary tract infections; however, the exact mechanism remains unclear [1,4,5,6,9]. The clinical presentation typically manifests as a combination of palpable purpura, abdominal pain, arthralgia/arthritis, and glomerulonephritis [1,2,3,5,6,7,9,10]. HSP is usually a self-limiting condition requiring supportive care with fluids, analgesics, and management of the underlying cause [1,3,5,7]. Medical management with corticosteroids or other immunosuppressants, as well as surgical management, should only be considered for severe cases [1,3,5,7].

*Giardia lamblia* (also referred to as *G. duodenalis* or *G. intestinalis*) is a microscopic parasite that primarily affects the gastrointestinal tract and is transmitted through the fecal–oral route [11,12,13,14]. The clinical presentation of giardiasis is subclinical or appears as acute or chronic gastroenteritis; post-infectious sequelae, either in the gastrointestinal tract or other organs, may occur [12,13,14,15]. This research paper provides novel insights into the association between HSP and parasites. The case report focuses on an unusual case, which is the first case in Greece, of an 8-year-old female with HSP secondary to *Giardia lamblia* infection. It highlights that strong clinical suspicion, like in this case, will lead to rapid diagnosis and effective treatment. The literature review focuses on this rare and underreported post-infectious complication, giving robust evidence on the clinical features, diagnosis, and treatment of HSP secondary to parasitic infections [9,16,17,18,19,20,21,22,23,24,25].

## 2. Case Presentation

**Figure 1:**
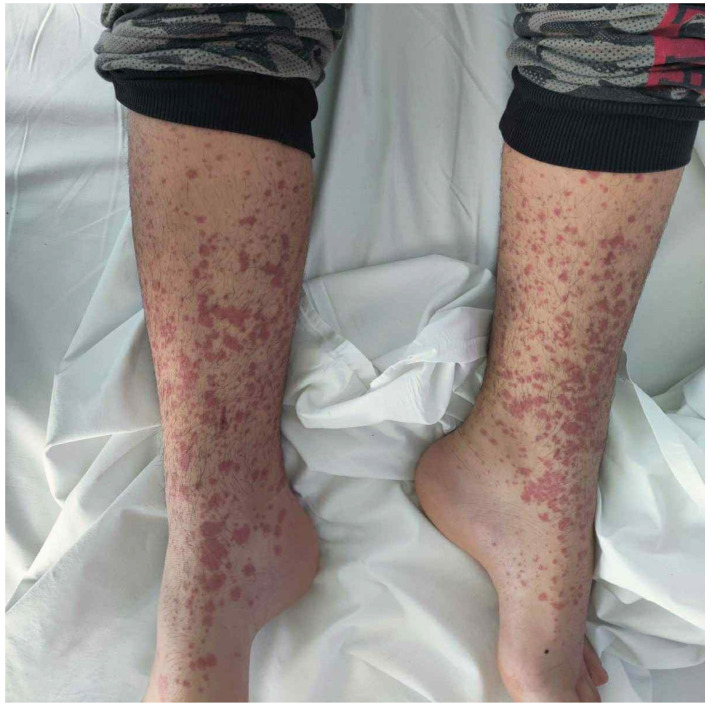
The morphology of the developed rash; note the presence of papules coalescing as a purpuric rash.

An 8-year-old female patient from the Roma ethnic group with an unremarkable medical history presented with a purpuric rash initially on the lower limbs and buttocks, which later spread to the abdomen and upper limbs (Figure 1). The knees, wrists, and distal malleolar regions were tender. She experienced severe, intermittent, diffuse abdominal pain unresponsive to acetaminophen for 2 days, followed by bloody diarrhea. There was no recent history of infections.

The patient’s medical and perinatal history was unremarkable. She was born full-term via cesarean section after an uneventful pregnancy. There was no history of chronic illness, hospitalizations, surgeries, or allergies. She had normal development.

The vital signs were within the normal range. Physical examination revealed a symmetrical purpuric and petechial rash on the buttocks and extremities. Joint tenderness was also present in the lower limbs during passive and active joint movements, as well as non-pitting edema in the knees and in the distal radial and malleolar regions. Abdominal examination revealed mild tenderness without rebound or guarding, normal bowel sounds, and a lack of organomegaly. The head and neck, chest, and neurological examination were unremarkable.

Cell blood count and comprehensive metabolic panel were within the normal range for her age. Serological tests for common viruses and rheumatic diseases were normal, except for an elevated IgA level [Table 1]. Urine analysis was normal, with no evidence of hematuria or proteinuria. Stool analysis was positive for *Giardia lamblia* antigen on two consecutive samples. Stool and upper respiratory cultures were negative. Abdominal ultrasound was unremarkable.

Upon admission, the patient received intravenous fluids, prednisolone (2 mg/kg/day), and omeprazole due to severe abdominal pain and bloody stools. After the positive antigen testing for *Giardia lamblia*, a 7-day regimen of metronidazole was administered. A gradual resolution of the patient’s symptoms was observed during the inpatient course. She was discharged after 5 days with a 2-week taper of prednisolone. Follow-up after 1 month revealed complete recovery and negative stool test results.

## 3. Discussion

### 3.1. Literature Review

Our literature search of PubMed and Scopus (June 2025) used the search term (IgA vasculitis OR HSP OR Henoch–Schönlein purpura) combined with each parasite name listed on the official website of the United States Centers for Disease Control and Prevention (CDC). We included English-language articles describing cases of HSP secondary to parasitic infections in pediatric patients. The exclusion criteria were non-English literature, patients older than 18 years, and HSP caused by non-parasitic agents. A total of eleven articles met the inclusion criteria, reporting 44 cases [Table 2]. Among these, 15 cases involved *Giardia lamblia*, 6 involved *Trichomonas hominis*, 5 involved *Entamoeba histolytica*, 2 involved *Strongyloides stercoralis*, 2 involved *Ascaris lumbricoides*, 1 involved *Diploscapter coronata*, 1 involved *Sarcoptes scabiei*, 1 involved *Toxoplasma gondii*, 1 involved *Plasmodium falciparum*, and 1 involved *Toxocara canis*. Of the 44 patients, 30 were males and 14 were females. Reported ages ranged from 2 to 17 years.

### 3.2. Correlation with Literature

Giardiasis is the most common parasitic disease and a major cause of infectious diarrhea in humans [11,12,13,14]. Waterborne (most common), foodborne, human, and animal transmissions are involved [11,12,13,14]. After ingestion, cysts convert into trophozoites in the small intestine, where they attach to the mucosa, causing immune-mediated disruption rather than invasion [11,12,13]. The trophozoites then detach and transform into cysts, which are excreted in the stool [11,12,13]. The involvement of the intestinal mucosa can be acute or chronic, leading to gastroenteritis, malabsorption, growth and cognitive impairments, and hypokalemic myopathy [11,12,13,14,15]. Persistent inflammation may cause post-infectious functional disorders like dyspepsia and irritable bowel syndrome [11,13,15]. Extra-intestinal complications, such as reactive arthritis, hypersensitivity reactions, chronic fatigue syndrome, and ocular disease, may result from systemic immune activation, even though *Giardia lamblia* cannot spread through the bloodstream [11,12,13,14,15]. Preventing similar cases of giardiasis is essential. It is of the utmost importance to use soap and water or antiseptics, especially during urination, defecation, and food preparation [12,14]. Purifying the water, preparing the food, avoiding overcrowding, and limiting animal contact are also important [12,13,14,15]. The application of these measures in day-care centers or swimming activities could limit the transmission of giardiasis in children and decrease the incidence of post-infectious complications [12,14]. HSP is a rarely reported immunological complication of *Giardia lamblia* infection in children, as shown in this review [16,17].

The standard diagnostic test is stool microscopy, while antigen or molecular testing are newer methods [14]. Repeating stool testing is often necessary [12]. Occasionally, diagnosis is established through a fluid aspirate or biopsy from the duodenum [14]. Oral use of an antimicrobial agent and a probiotic, e.g., *Lactobacillus* spp., along with rehydration and nutritional support, is essential [11,14,26,27]. First-line treatments include nitroimidazoles, such as metronidazole and tinidazole [11,12,14]. Alternative options include benzimidazoles (albendazole and mebendazole), nitazoxamide, paromomycin, acridine, furazolidone, and chloroquine [12,14]. Critical illness or immunosuppression are contraindications for treatment with probiotics [14,26]. In this case, antigen testing confirmed the diagnosis, followed by metronidazole therapy. Probiotics were avoided due to gastrointestinal bleeding and glucocorticoid treatment.

HSP is multi-organ vasculitis resulting from the deposition of IgA immune complexes (ICs) in the microcirculation and the most common vasculitis in pediatric patients [1,3,4,5,7,9]. Risk factors include an age of less than 10 years, male sex, white/Asian race, family history of HSP, and a past medical history of familial Mediterranean fever [1,4,5,6,7,8,16]. Infections are considered the most common trigger of HSP; this fact explains the decrease in incidence during summer [1,4,5,6,7,9,16]. Drugs, vaccines, insect bites, dietary allergens, and neoplasms have been reported as causes of HSP [4,6,10,16,23,24]. The most relevant pathogens are viral and bacterial pathogens [1,4,9,16]. Common examples include coronavirus, parvovirus B19, varicella, rubella, measles, mumps, coxsackie, adenovirus, parainfluenza, respiratory syncytial virus, hepatitis A, hepatitis B, human immunodeficiency virus, *Streptococcus*, *Staphylococcus*, *Salmonella*, *Bartonella henselae*, *Mycoplasma pneumoniae*, *Helicobacter pylori*, *Salmonella*, *Clostridium*, and tuberculosis [1,9,18,28]. Parasites, such as *Giardia lamblia* in our case, are responsible for a minority of pediatric HSP cases, as shown by the literature review [16,17,27,29].

The pathogenesis of HSP is incompletely understood; genetic background and environmental factors are considered essential, as in IgA nephropathy [1,4,5,7,10,16,24]. A plausible mechanism suggests that activated Th2 cells secrete interleukin-6 (IL-6), which induces the production of galactose-deficient IgA1 (Gd-IgA1) [4,5,24]. This leads to tissue deposition of ICs, activation of complement, and inflammatory damage [4,5,6,16,24]. Another theory suggests that molecular mimicry triggers the formation of IgA1 anti-endothelial cell antibodies (AECAs) [4,6]. AECAs target the β2 glycoprotein I receptor on endothelial cells; thus, IL-8 is released, leading to neutrophil infiltration of the microcirculation [4,6].

Possible mechanisms of extraintestinal complications in giardiasis include impairment of the intestinal barrier, dissemination of parasitic toxins or food allergens in the systemic circulation, and tissue deposition of antigens from enteric pathogens [12,13,15]. Notably, humoral immunity is activated after innate immunity to eliminate the parasite [11]. Although the pathogenesis of HSP in the context of giardiasis remains unclear, mucosal infections may trigger the differentiation of B-cells into IgA-producing plasma cells, which is the initial step in this post-infection sequela [27]. It could also be hypothesized that antigens leak into systemic circulation due to intestinal disruption, bind IgA molecules, and promote the formation of ICs [6,12,13,15]. Co-infections may be more likely to cause HSP than isolated giardiasis [27]. Establishing a direct causal relationship between giardiasis and HSP remains challenging due to the multifactorial etiology of IgA vasculitis. Genetic and environmental factors, including multiple pathogens, are all known to contribute to the disease [1,4,5,6,9,16]. Hence, the role of each pathogen is often unclear [1,4,5,6,9,16]. In addition, infectious diseases commonly precede HSP by days to weeks and may be asymptomatic [1,11,12,13,14,15]. Moreover, HSP affects the gastrointestinal tract, predisposing it to superimposed infections [18,20,21]. Thus, the presence of parasites may be either a precipitating factor or a coincidental finding. Further studies in basic research (in vitro experiments and animal models) and clinical research (cohort and case-control studies) could clarify the immunopathogenesis and the causal relationship between parasitic infections and HSP.

The clinical presentation involves multiple organs, but skin involvement is universal [1,2,4,5,7,9,16,18,27]. Rash is defined as palpable purpura and petechiae, mostly in the lower limbs and buttocks, while angioedema may be present [1,2,5,9,16,27]. Atypical cases of rash, such as those with plantar distribution, have been reported in the literature [2,16]. Non-deforming arthritis or arthralgia with knee and ankle predominance is present in most cases [1,2,5,9,16]. Diffuse and colicky abdominal pain with nausea, vomiting, and diarrhea is common [1,2,5,9,16]. Rare but severe complications include gastrointestinal bleeding, intussusception, appendicitis, and bowel perforation [1,2,5,9,16]. Renal involvement presents as hematuria, proteinuria, and hypertension; nephritic syndrome, nephrotic syndrome, and end-stage renal disease represent major complications of HSP [1,2,3,4,7,9,16]. Coagulopathy, testicular, respiratory, neurological, cardiac, and liver involvement are unusual [1,3,5,7,16]. The timing of the symptoms and signs of HSP is variable, and their complete development might occur after a few days or weeks [1,18]. In this case, the diagnosis was straightforward given the combination of rash, arthralgia, and abdominal pain.

The diagnosis of HSP is primarily clinical, although laboratory tests detect potential complications and exclude similar diseases [1,3,5,16,30]. A minimum diagnostic work-up includes cell blood count, coagulation profile, basic metabolic panel, urinalysis, fecal occult blood test, albumin, and blood/urine cultures [1,3,5,16,30]. Additional tests for the investigation of post-streptococcal complications and rheumatic diseases include antistreptolysin O (ASTO), antideoxyribonuclease B (anti-DNAse B), antinuclear antibodies (ANA), anti-double-strand deoxyribonucleic acid (anti-dsDNA), cellular antineutrophil cytoplasmic antibodies (c-ANCA), perinuclear antineutrophil cytoplasmic antibodies (p-ANCA), immunoglobulins, complement, and angiography [7,16]. Imaging (testicular ultrasound, abdominal ultrasound, neuroimaging) and endoscopy of the gastrointestinal or respiratory tract may be necessary for investigating organ-related complications [1,3,7,16,31]. Skin biopsy is indicated in equivocal cases and reveals the deposition of IgA and leukocytoclastic vasculitis [1,3,5,7,28]. Renal biopsy is necessary for the stratification of severe kidney involvement; it reveals findings similar to IgA nephropathy, such as mesangial proliferation and IgA deposition [1,3,7,16,28]. In this case, appropriate laboratory and imaging testing excluded similar diseases, and a skin biopsy was unnecessary given the classic clinical findings.

The differential diagnosis in a pediatric patient with rash and systemic symptoms requires careful consideration of similar diseases [30]. A normal CBC and the lack of lymphadenopathy rule out thrombocytopenic purpura and leukemic infiltration [5,16,30]. The absence of signs of sepsis, such as fever, hypotension, and coagulopathy, rules out meningococcemia [16,30,32,33,34]. Viral and rickettsial exanthematous diseases are important infectious causes of febrile rash that should be considered [30,33,35,36]. The lack of fever, valvular involvement, septic emboli, Janeway lesions, and Osler nodes is inconsistent with infective endocarditis [16]. The absence of recent streptococcal infection, carditis, migratory arthritis, chorea, and erythema marginatum excludes the diagnosis of acute rheumatic fever [16,35,36,37]. Inflammatory bowel disease is more likely to present during adolescence or young adulthood with chronic diarrhea, weight loss, axial spondylarthritis, erythema nodosum, or pyoderma gangrenosum [30]. Serum sickness and serum sickness-like reaction are associated with recent drug exposure and present with urticarial, erythema multiforme-like, or maculopapular rash [35,36,37,38]. However, gastrointestinal involvement is less prominent [36]. Juvenile idiopathic arthritis typically presents with a salmon-like rash, fever, and uveitis, requiring a minimum of six weeks for diagnosis [30,36,38]. The absence of fever, cervical lymphadenopathy, mucositis, palmoplantar rash, and cardiac involvement rules out Kawasaki disease [30,33,36,38]. Polyarteritis nodosa typically presents as a chronic illness in middle-aged male patients [5,16,30]. The lack of antineutrophil cytoplasmic antibodies (ANCA), respiratory, and renal involvement excludes the diagnosis of granulomatosis with polyangiitis [5,30].

HSP is considered self-limiting, with a favorable prognosis [1,3,5,7,9]. Treatment is primarily symptomatic with fluids and analgesics, such as acetaminophen or non-steroidal anti-inflammatory agents (NSAIDs) [1,3,5,7,9]. However, renal damage or gastrointestinal bleeding is a contraindication for the administration of NSAIDs [1,3,5,7,9]. Corticosteroids and non-steroidal immunosuppressants should be administered under specific circumstances for the management of severe complications [1,5,7,9]. It should be noted that immunosuppressants, such as corticosteroids, should be used cautiously because they can worsen the severity of an underlying parasitic infection [18,22,24]. Thus, the management of the underlying cause, such as infections, is essential [7,9,29]. However, the use of antibiotics should be justified, given their potential association with an exacerbation of HSP [21]. In this case, intravenous fluids and corticosteroids were preferred over NSAIDs due to gastrointestinal bleeding, while metronidazole successfully treated giardiasis.

## 4. Conclusions

Pediatricians should be aware of the rare association between Giardiasis and HSP, as presented in this research paper. Given the overlapping gastrointestinal symptoms, a strong clinical suspicion is essential to initiating the appropriate investigation. Early diagnosis and treatment of the underlying Giardiasis might accelerate the resolution of post-infectious sequelae.

## Figures and Tables

**Table 1:** Laboratory test results of this case. All findings were normal, except for an elevated IgA level.

**Serologic Tests (AU/mL)**	
EBV IgM: 0.22 (<1)	EBV IgG: 40.8 (<1)
CMV IgM: 10.4 (<22)	CMV IgG: 141 (<14)
Parvovirus B19 IgM: < 0.1 (<1)	Parvovirus B19 IgG: <0.1 (<1)
**Immunoglobulins**	
IgA (mg/dL): 287 (45–236)	IgE (IU/mL): 119 (5–165)
IgG (mg/dL): 1680 (670–1590)	IgM (mg/dL): 96 (46–304)
Globulins (mg/dL): 3400 (2000–3500)	
**Vasculitis screening**	
C3 (mg/dL): 152 (79–153)	C4 (mg/dL): 49 (10–40)
c-ANCA: negative	p-ANCA: negative
CRP (mg/dL): 1.64 (<0.8)	ANA: negative
ASTO (IU/mL): 535 (<200)	
**Microbial screening**	
Blood culture: negative	Throat culture: negative
Stool culture: negative	Stool analysis: positive antigen for *Giardia lamblia*
**Complete blood count**	
Hemoglobin (g/dL): 12	Hematocrit: 36.1
White blood cells (K/μL): 7.62	Neu/Lymph/Mon/Eos/Baso (%): 80.2/18.8/1/0.1/0
Platelets (K/μL): 410	
**Comprehensive metabolic panel (CMP)**	
Sodium (mmol/L): 137	Potassium (mmol/L): 4.5
Calcium (mg/dL): 10	Glucose (mg/dL): 128
Creatinine (mg/dL): 0.5	Urea (mg/dL): 25
Albumin (g/dL): 4.5	Total Protein (g/dL): 7.9
AST (U/L): 17	ALT (U/L): 13
Alkaline Phosphatase (U/L): 210	γ-GT (U/L): 11

Abbreviations: EBV: Epstein-Barr virus, CMV: Cytomegalovirus, IgM: Immunoglobulin M, IgG: Immunoglobulin G, IgA: Immunoglobulin A, IgE: Immunoglobulin E, c-ANCA: c-antineutrophil cytoplasmic antibody, p-ANCA: p-antineutrophil cytoplasmic antibody, CRP: C-reactive protein, ANA: antinuclear antibodies, ASTO: antistreptolysin O, Neu: neutrophils, Lymph: lymphocytes, Mon: monocytes, Eos: eosinophils, Baso: basophils, AST: aspartate aminotransferase, ALT: Alanine Aminotransferase, γ-GT: gamma-glutamyl transferase.

**Table 2:** Reported cases of HSP secondary to parasitic infections in the pediatric population.

Reference	Number of Patients	Age	Parasitic Agent	Sex	Clinical Presentation	Treatment
Causey et al., 1994, United States of America (Caucasian) [16]	1	28 months	*Giardia lamblia*	male	Anorexia, vomiting, abdominal pain, fever, and petechial rash	Intravenous fluids, furoxone, and corticosteroids
Kim et al., 2010, Korea [18]	1	8 years	*Entamoeba histolytica*	female	Abdominal pain, watery diarrhea, anorexia, arthritis, and purpuric rash	Metronidazole, and corticosteroids
Morimoto et al., 2006, Japan [19]	1	8 years	*Diploscapter coronata*	female	Arthralgia, purpuric rash, earache, abdominal pain, and constipation	Cefcapene followed by dexamethasone and carbazochrome sodium sulphonate
Demircin et al., 1998, Turkey [20]	1	11 years	*Entamoeba histolytica*	male	Fever, abdominal pain, myalgia, weight loss, maculopapular rash, arthritis, hematuria, and melena	Prednisolone and metronidazole
Nakandakari et al., 2021, Peru [21]	1	4 years	*Strongyloides stercoralis*	female	Dry cough and rhinorrhea followed by fever, epigastralgia, maculopapular/purpuric rash, and hematemesis	Metamizole and piperazine followed by a liquid diet, intravenous fluids, ceftriaxone, metronidazole, ivermectin, omeprazole, and finally dexamethasone
Wu et al., 2021, China [22]	1	11 years	*Sarcoptes scabiei*	male	Pruritic papular rash followed by purpuric rash and abdominal pain	Permethrin lotion, cetirizine, corticosteroids, and mycophenolate mofetil
Wang et al., 2020, China [9]	1	Not applicable	*Toxoplasma gondii*	Not applicable	Not applicable	Not applicable
Janković et al., 2016, Serbia [24]	1	14 years	*Strongyloides stercoralis*	female	Macular/purpuric rash, vomiting, abdominal pain, arthralgia, hypertension, and melena	Corticosteroids, antihistamine, hypoallergenic diet, mebendazole, azathioprine, and enalapril
Thapa et al., 2010, United States of America [23]	1	9 years	*Plasmodium falciparum*	male	Fever, anorexia, vomiting, hypotension, hyperreflexia, hepatosplenomegaly, altered mental status, and seizures followed by macular/purpuric rash, abdominal pain, and hematochezia	Artesunate, antiepileptics, transfusion, and primaquine
Ergür et al., 1999, Turley [17]	35	2–15 years	*Giardia lamblia* (*n =* 14) *Trichomonas hominis* (*n* = 6) *Entamoeba histolytica* (*n =* 3) *Ascaris lumbricoides* (*n* = 2)	25 males and 10 females	purpuric rash (*n* = 35) gastrointestinal involvement (*n* = 28) arthralgia/arthritis (*n* = 12) renal involvement (*n* = 8) scalp edema (*n* = 2) encephalopathy (*n* = 1)	Not applicable
Hamidou et al., 1999, France [25]	1	17 years	*Toxocara canis*	male	Purpuric rash, arthritis, and abdominal pain	Not applicable
Our case	1	8 years old	*Giardia lamblia*	female	Rash, arthralgia, abdominal pain, and bloody diarrhea	Intravenous fluids, prednisolone, omeprazole, metronidazole

## Data Availability

The original contributions presented in this study are included in this article; further inquiries can be directed to the corresponding author.

## Abbreviations

The following abbreviations are used in this manuscript:

AECA	Anti-endothelial cell antibody
ALT	Alanine aminotransferase
ANA	Antinuclear antibodies
ANCA	Antineutrophil cytoplasmic antibody
antiDNAse B	Antideoxyribonuclease B
AST	Aspartate aminotransferase
ASTO	Antistreptolysin O
Baso	Basophils
c-ANCA	Antineutrophil cytoplasmic antibodies
C3	Complement components 3
C4	Complement components 4
CBC	Complete blood count
CDC	Centers for Disease Control and Prevention
CMP	Comprehensive metabolic panel
CMV	Cytomegalovirus
CRP	C-reactive protein
EBV	Epstein–Barr virus
Eos	Eosinophils
Gd-IgA1	Galactose-deficient Immunoglobulin A1
HIV	Human Immunodeficiency Virus
HSP	Henoch–Schönlein purpura
IC	Immune complex
IgA	Immunoglobulin A
IgAV	IgA vasculitis
IgE	Immunoglobulin E
IgG	Immunoglobulin G
IgM	Immunoglobulin M
IL-6	Interleukin 6
IL-8	Interleukin 8
Lymph	Lymphocytes
Mon	Monocytes
Neu	Neutrophils
NSAIDs	Non-steroidal anti-inflammatory drugs
p-ANCA	Perinuclear antineutrophil cytoplasmic antibodies
Th2	T-helper cell type 2
γ-GT	Gamma-glutamyl transferase

## References

[1] Reamy B.V., Servey J.T., Williams P.M. (2020). Henoch-Schönlein Purpura (IgA Vasculitis): Rapid Evidence Review. Am. Fam. Physician.

[2] Ozen S., Pistorio A., Iusan S.M., Bakkaloglu A., Herlin T., Brik R., Buoncompagni A., Lazar C., Bilge I., Uziel Y. (2010). EULAR/PRINTO/PRES criteria for Henoch–Schönlein purpura, childhood polyarteritis nodosa, childhood Wegener granulomatosis and childhood Takayasu arteritis: Ankara Part II: Final classification criteria. Ann. Rheum. Dis..

[3] Ozen S., Marks S.D., Brogan P., Groot N., de Graeff N., Avcin T., Bader-Meunier B., Dolezalova P., Feldman B.M., Kone-Paut I. (2019). European consensus-based recommendations for diagnosis and treatment of immunoglobulin A vasculitis—The SHARE initiative. Rheumatology.

[4] Xu L., Li Y., Wu X. (2022). IgA vasculitis update: Epidemiology, pathogenesis, and biomarkers. Front. Immunol..

[5] Castañeda S., Quiroga-Colina P., Floranes P., Uriarte-Ecenarro M., Valero-Martínez C., Vicente-Rabaneda E.F., González-Gay M.A. (2024). IgA Vasculitis (Henoch-Schönlein Purpura): An Update on Treatment. J. Clin. Med..

[6] Song Y., Huang X., Yu G., Qiao J., Cheng J., Wu J., Chen J. (2021). Pathogenesis of IgA Vasculitis: An Up-To-Date Review. Front. Immunol..

[7] Abu-Zaid M.H., Salah S., Lotfy H.M., El Gaafary M., Abdulhady H., Tabra S.A.A., Salah H., Farag Y., Eissa M., Maher S.E. (2021). Consensus evidence-based recommendations for treat-to-target management of im-munoglobulin A vasculitis. Ther. Adv. Musculoskelet. Dis..

[8] Gardner-Medwin J.M., Dolezalova P., Cummins C., Southwood T.R. (2002). Incidence of Henoch-Schonlein purpura, Kawasaki disease, and rare vasculitides in children of different ethnic origins. Lancet.

[9] Wang J.J., Xu Y., Liu F.F., Wu Y., Samadli S., Wu Y.F., Luo H.H., Zhang D.D., Hu P. (2020). Association of the infectious triggers with childhood Henoch–Schonlein purpura in Anhui province, China. J. Infect. Public Health.

[10] Rasmussen C., Tisseyre M., Garon-Czmil J., Atzenhoffer M., Guillevin L., Salem J.-E., Treluyer J.-M., Terrier B., Chouchana L. (2021). Drug-induced IgA vasculitis in children and adults: Revisiting drug causality using a dual pharmacovigilance-based approach. Autoimmun. Rev..

[11] Einarsson E., Ma’ayeh S., Svärd S.G. (2016). An up-date on Giardia and giardiasis. Curr. Opin. Microbiol..

[12] Leung A.K.C., Leung A.A.M., Wong A.H.C., Sergi C.M., Kam J.K.M. (2019). Giardiasis: An Overview. Recent Patents Inflamm. Allergy Drug Discov..

[13] Allain T., Buret A.G. (2020). Pathogenesis and post-infectious complications in giardiasis. Adv. Parasitol..

[14] Shane A.L., Mody R.K., Crump J.A., Tarr P.I., Steiner T.S., Kotloff K., Langley J.M., Wanke C., Warren C.A., Cheng A.C. (2017). 2017 Infectious Diseases Society of America Clinical Practice Guidelines for the Diagnosis and Management of Infectious Diarrhea. Clin. Infect. Dis..

[15] Halliez M.C.M., Buret A.G. (2013). Extra-intestinal and long term consequences of Giardia duodenalis infections. World J. Gastroenterol..

[16] Causey A.L., Woodall B.N., Wahl N.G., Voelker C.L., Pollack E.S. (1994). Henoch-Schönlein purpura: Four cases and a review. J Emerg. Med..

[17] Ergür A.T., Cetinkaya O., Onarlioğlu B. (1999). Paediatric patients with Henoch-Schönlein purpura followed up at Cumhuriyet Uni-versity, Sivas, Turkey during 1993–1996: Role of parasitosis in the aetiology of Henoch-Schönlein purpura. J. Trop. Pediatr..

[18] Kim Y.O., Choi Y.S., Won Y.H., Kim Y.D., Woo Y.J., Back H.J., Cho Y.K., Han D.K., Song E.S. (2010). Intestinal amebiasis with Henoch-Schönlein purpura. Pediatr. Int..

[19] Morimoto N., Korenaga M., Yagyu K., Kagei N., Fujieda M., Bain O., Wakiguchi H., Hashiguchi Y., Sugiura T. (2006). Morphological observations and the effects of artificial digestive fluids on the survival of *Diploscapter coronata* from a Japanese patient. J. Helminthol..

[20] Demircin G., Oner A., Erdoğan O., Bülbül M., Memiş L. (1998). Henoch Schönlein purpura and amebiasis. Acta Paediatr..

[21] Nakandakari Gomez M.D., Marín Macedo H., Seminario Vilca R. (2021). IgA (Henoch Schönlein Purpura) Vasculitis in a Pediatric Patient with COVID-19 and Strongyloidiasis. Rev. Fac. Med. Humana.

[22] Wu Y.F., Wang J.J., Liu H.H., Chen W.X., Hu P. (2021). Scabies, incomplete lupus erythematosus and Henoch-Schonlein purpura. Arch. Med. Sci..

[23] Thapa R., Mallick D., Biswas B., Ghosh A., Chakrabartty S., Dhar S. (2010). Henoch-Schönlein Purpura Triggered by Falciparum Cerebral Malaria. Clin. Pediatr..

[24] Janković S., Nikolić M., Simović A., Vujić A. (2016). Henoch-Schönlein purpura associated with *Strongyloides stercoralis* infection. Vojnosanit. Pregl..

[25] Hamidou M.A., Gueglio B., Cassagneau E., Trewick D., Grolleau J.Y. (1999). Henoch-Schönlein purpura associated with *Toxocara canis* infection. J. Rheumatol..

[26] Kapnisis D., Chatzigrigoriadis C., Koufopoulos E., Kolonitsiou F., Dimitriou G., Fouzas S., Eskitzis P., Lavasidis L., Anestakis D., Sperdouli D. (2025). An Unusual Cause of Neonatal Infection: A Case Report of *Campylobacter coli* Meningitis and Sepsis. J. Med. Cases.

[27] Castellino J., Orentas M., Hassan D., Khandelwal S. (2023). IgA Vasculitis in an Adult Linked to Cryptosporidium and Giardia Co-Infection: A Comprehensive Case Study. Am. J. Case Rep..

[28] Mohamed M., Shariff M., Al Hillan A., Al Haj R., Kaunzinger C., Hossain M., Asif A., Pyrsopoulos N.T. (2020). A Rare Case of *Helicobacter pylori* Infection Complicated by Henoch-Schonlein Purpura in an Adult Patient. J. Med. Cases.

[29] Mastroianni A., Greco S., Vangeli V., Mauro M.V., Greco F., Manfredi R. (2023). Lambliasis-associated Schonlein-Henoch purpura in an Italian traveller: First case report in Italy. Int. Marit. Health.

[30] Reamy B.V., Williams P.M., Lindsay T.J. (2009). Henoch-Schönlein purpura. Am. Fam. Physician.

[31] Zhang F., Chen L., Shang S., Jiang K. (2018). Atypical purpura location in a pediatric patient with Henoch-Schönlein purpura: A case report. Medicine.

[32] Filippakis D., Gkentzi D., Dimitriou G., Karatza A. (2020). Neonatal meningococcal disease: An update. J. Matern. Fetal. Neonatal Med..

[33] Muzumdar S., Rothe M.J., Grant-Kels J.M. (2019). The rash with maculopapules and fever in children. Clin. Dermatol..

[34] Poulikakos P., Kapnisis D., Xirogianni A., Liakou I., Tsolia M., Michos A., Mantadakis E., Papaevangelou V., Iliadis A., Gkentzi D. (2025). Invasive Meningococcal Disease in Children: Outcomes and Risk Factors for Sequelae and Fatal Cases in Greece. Microorganisms.

[35] Chatzigrigoriadis C., Eleftherakis G., Gyftopoulos K., Assimakopoulos S.F. (2025). To Test or Not to Test? How a Positive Rapid Strep Test May Perplex the Diagnosis of Serum Sickness-Like Reaction in a Case Report. Int. J. Med. Stud..

[36] Chatzigrigoriadis C., Koufopoulos E., Avramidis P., Erginousakis I., Karakoida V., Papadopoulos T., Sperdouli D., Tachliabouri M.-E., Vilanakis K., Zampounidis D. (2025). Serum Sickness-Like Reaction: A Narrative Review of Epidemiology, Immunopathogenesis, Diagnostic Challenges, and Therapeutic Approaches. Clin. Pract..

[37] Patterson-Fortin J., Harris C.M., Niranjan-Azadi A., Melia M. (2016). Serum sickness-like reaction after the treatment of cellulitis with amoxicillin/clavulanate. BMJ Case Rep..

[38] Sekerel B.E., Ilgun Gurel D., Sahiner U.M., Soyer O., Kocaturk E. (2023). The many faces of pediatric urticaria. Front. Allergy.

